# Neglected Advantages

**Published:** 1889-11

**Authors:** Albert H. Brockway


ARTICLE III.

### NEGLECTED ADVANTAGES.

BY ALBERT H. BROCKWAY, M. D. S.

[A paper read before the New York Odontological Society.]

No fact seems to be more generally admitted than that the present is an age of improvement. The triumphs of steam and electricity, whereby our ready communication and exchange are so vastly promoted, are fitly supplemented by the growth of more enlightened and liberal views in science, ethics, and religion, softening and broadening in their influence, and steadily tending to bring in peace and good-will among men. The laws of sanitary science are inquired into with a zeal born of enthusiasm, that the "pestilence that walketh in darkness and wasteth at noon-day" may be stayed. The reform in political methods is undertaken and carried forward by earnest and unselfish men to the end that good government and wise policy be promoted, and justice and prosperity prevail.

But no observant person can fail to be struck with the fact that, after all, the acceptance of new ideas and new methods is of really slow growth, limited at first to a few receptive minds; that the mass of mankind are not easily changed from the habits and methods to which they have become accustomed. To them appears as the highest wisdom the smug and satisfied philosophy embodied in the couplet:

"Be not the first by whom the new is tried,
Nor yet the last to lay the old aside."

forgetful that were it adopted by *all* there would be an end of progress.

Reflection upon this phase of the subject has led me to better understand what I confess has sometimes surprised me in the attitude held by so many in our own chosen profession, which is but an epitome and microcosm of the larger world.

That the improvements made within the past few years in the practice of dentistry—especially in the appliances used—have been very great, all will admit. We are, indeed, apt to think of our progress as phenomenal and exceeding that in most other branches of human activity, which is perhaps in consequence of our nearer view. Be that as it may, the fact remains that we have at our command in the present day many advantages in the conduct of our beneficent work not possessed or scarcely even dreamed of by our fathers—advantages which, if not neglected, but rightly employed, are capable of increasing our capacity and consequent usefulness to those under our care to a most marked degree. The statement does not readily admit of illustration by statistics, so much of the purely personal element is to be taken into the account, or I should be tempted to undertake it. I can only say, in passing, that in my individual experience as demonstrated by my recorded operations the benefit derived has been sufficient to more than double my capacity for work.

That many of these advantages are neglected by not a few among us, to their own and their patient's detriment, is to me therefore a matter of regret, and I shall endeavour in the short time at my disposal to designate a few of the more prominent improvements, and comment briefly upon their value, in the hope that thereby they may be more generally appreciated and made use of.

Not undertaking to indicate the order of importance, I shall speak of the assistant, the burring engine, the matrix and the separator.

And first of the assistant at the chair. There is, I am aware, in the minds of many dentists, a prejudice against the employment or presence even of another person at the operating chair,—a prejudice which I confess I myself once shared, but which experience has shown to be unfounded and absurd to the last degree. They fancy that the presence of another will in a measure interfere with the somewhat personal and confidential relation which the dentist should hold toward his patient, and that fastidious patients might object to their employment. Granting the objection its full weight, I am convinced that it should not be for a moment considered in view of the advantages which can be set against it. To the operator aided by a trained and intelligent assistant is given another pair of hands—and sometimes we fancy that Briareus with his hundred arms would have too few for the emergency!—another pair of eyes to look for the elusive and particular instrument required, two willing feet to fetch the needed article just out of reach, and another brain to take thought of the order and care of the surroundings. To the patient is given the service of one less preoccupied than the principal, to see to his needs, look after his comfort, and help in a hundred nameless ways to shorten the tedium and fatigue of the dreaded sitting.

Objection is sometimes made to the employment of an assistant on the ground of expense, many declaring that they cannot afford it, seeming to regard it in the nature of a needless luxury; and I have, in advocating this advantage, not infrequently been asked what use I could find for an assistant aside from malleting.

The objection of expense can be urged against the employment of any means of improvement; but expense is relative, and what seems needless extravagance may in fact be the wisest economy. No one, however limited his practice, can afford to forego the advantage of an assistant; on the contrary, the increased efficiency and celerity which he thereby secures—to say nothing of the added comfort which is insured to his patient—will repay tenfold the probable expense incurred.

A word as to what kind of assistant. Having tried them of both kinds, I give the preference to those of the gentler sex, as being, all things considered, better adapted by nature to fill the office; and to illustrate; I will mention some of the services rendered me by the young lady who has for some time past been associated with me in this capacity.

In the first place, she looks to the general condition of the fixtures of the office after the servants have properly swept and dusted, to see that everything is in proper order for the reception of patients; she keeps in condition the instruments and appliances used, bringing them to me as wanted and returning them to place when done with; she prepares in advance all articles that may be needed, such as absorbent paper, pellets, waxed ligatures for adjusting the rubber-dam, swabs for dressing root-canals; she assists in putting on the rubber-dam, or in using the separator or matrix; she holds away the lips or the tongue of the patient when needed, to avoid abrasion or secure a better view; prepares ready to my hand the materials for filling, and assists in packing them into the cavity, and renders a thousand and one little services to the saving of my time and strength and the preservation of my good temper that time would fail me to enumerate.

The number among us who fail to make use of the burring-engine in some measure is perhaps too small to be seriously considered, yet I am of opinion that not a few fail to get from it all the advantage which they might. The operator who wastes his strength and sacrifices in some degree his steadiness of touch by driving his own engine, standing on one leg, stands no less in his own light. This matter should be delegated to an assistant, or, better still, some motor power should be employed for the purpose. There can be little excuse at the present day for not doing so. I have in my own practice for several years made use of a water motor, put in at an expense of about one hundred dollars, and costing me in taxes some eight dollars yearly, which I am confident has been a most profitable investment. The steadiness of the bur when driven by a uniform power makes its use less painful than when driven in the usual way by the treadle, besides the operator and assistant are left free to give their attention to other matters. But great as is the advantage secured by the burring engine in the preparation of ordinary and accessible cavities, in reaching those upon the posterior surfaces of teeth far back in the mouth it is *indispensable*, if time and the feelings of the patient are to be taken into account. By the aid of the corundum-stone and the back-action hand-piece, such cavities can be readily exposed and prepared with nearly as much facility as any; while with the help of a suitable *matrix* the introduction of a proper filling is rendered far less difficult and uncertain than would otherwise be possible.

It is in such cases as these, and in those more accessible teeth where considerable loss of substance has taken place, that I find most use for the matrix in some form; and while I do not myself make use of it in filling many approximal cavities in the molars and bicuspids, I have been surprised to hear, within a short time past, two deservedly prominent dentists admit that they had never even *tried* such an appliance. However, I am satisfied that the matrix is too useful to be wholly discarded, though less a necessity than some other things.

The separator, as introduced in its crude form by the late Dr. Jarvis and modified by Dr. Perry and Dr. Bogue, also the forms devised by Dr. Parr, Dr. Elliott, and others, has met with a less universal acceptance than its great merit demands. It is one of the most useful devices for saving time, both to the operator and patient, that has been introduced. Nor is time alone saved, but pain and discomfort as well. By its employment not only are the teeth separated quickly without serious discomfort, but being also thereby held firmly in the changed position their sensibility is greatly reduced, as every one familiar with the use of this instrument has doubtless observed. I am aware that many quite progressive dentists have not yet availed themselves of the manifest advantage of the separator from fear that injury might be done by its use, or that it would provoke protests from their patients. This objection might be urged against almost any instrument we have, and with about as much force. I have never known of harm done by its use, while we are all familiar with cases where serious mischief has been wrought through the careless separation of teeth by the ordinary methods with wedges of rubber, wood or cotton.

It is true that the sensation caused by the application of the separator is at the first somewhat unpleasant, possibly painful; but it is only momentary, and patients after experience with it almost universally express their preference for it over other means. The time saved by its use is no small item.

To apply it will take, let us say, at a liberal estimate, perhaps five minutes in the most difficult case, and of course much less usually. By the ordinary method of separation nearly as much time will be consumed in getting the patient into the chair and introducing a wedge of cotton or rubber, which not infrequently will require one or more renewals before sufficient space is gained, each renewal taking a few moments of the dentist's time and involving another visit on the part of the patient. In a full practice the aggregate waste of time is a serious loss.

But as I did not set out to cover the whole field of neglected advantages, I will not further continue. In what has been said, my desire has been to impress upon such of my professional brethren as have for any reason failed to fully appreciate the benefits of the appliances mentioned my own belief in their value, and to ask them seriously to consider whether they can afford to forego any advantages of this nature which are within their reach. If any shall be aided in this by what has been said, I shall feel that I have not spoken in vain.—*The Dental Cosmos.*

